# First Experimental Demonstration of the Multipotential Carcinogenic Effects of Aspartame Administered in the Feed to Sprague-Dawley Rats

**DOI:** 10.1289/ehp.8711

**Published:** 2005-11-17

**Authors:** Morando Soffritti, Fiorella Belpoggi, Davide Degli Esposti, Luca Lambertini, Eva Tibaldi, Anna Rigano

**Affiliations:** Cesare Maltoni Cancer Research Center, European Ramazzini Foundation of Oncology and Environmental Sciences, Bologna, Italy

**Keywords:** artificial sweetener, aspartame, carcinogenicity, lymphomas, malignant schwannomas, rats, renal pelvis carcinomas

## Abstract

The Cesare Maltoni Cancer Research Center of the European Ramazzini Foundation has conducted a long-term bioassay on aspartame (APM), a widely used artificial sweetener. APM was administered with feed to 8-week-old Sprague-Dawley rats (100–150/sex/group), at concentrations of 100,000, 50,000, 10,000, 2,000, 400, 80, or 0 ppm. The treatment lasted until natural death, at which time all deceased animals underwent complete necropsy. Histopathologic evaluation of all pathologic lesions and of all organs and tissues collected was routinely performed on each animal of all experimental groups. The results of the study show for the first time that APM, in our experimental conditions, causes *a*) an increased incidence of malignant-tumor–bearing animals with a positive significant trend in males (*p* ≤ 0.05) and in females (*p* ≤ 0.01), in particular those females treated at 50,000 ppm (*p* ≤ 0.01); *b*) an increase in lymphomas and leukemias with a positive significant trend in both males (*p* ≤ 0.05) and females (*p* ≤ 0.01), in particular in females treated at doses of 100,000 (*p* ≤ 0.01), 50,000 (*p* ≤ 0.01), 10,000 (*p* ≤ 0.05), 2,000 (*p* ≤ 0.05), or 400 ppm (*p* ≤ 0.01); *c*) a statistically significant increased incidence, with a positive significant trend (*p* ≤ 0.01), of transitional cell carcinomas of the renal pelvis and ureter and their precursors (dysplasias) in females treated at 100,000 (*p* ≤ 0.01), 50,000 (*p* ≤ 0.01), 10,000 (*p* ≤ 0.01), 2,000 (*p* ≤ 0.05), or 400 ppm (*p* ≤ 0.05); and *d*) an increased incidence of malignant schwannomas of peripheral nerves with a positive trend (*p* ≤ 0.05) in males. The results of this mega-experiment indicate that APM is a multipotential carcinogenic agent, even at a daily dose of 20 mg/kg body weight, much less than the current acceptable daily intake. On the basis of these results, a reevaluation of the present guidelines on the use and consumption of APM is urgent and cannot be delayed.

Consumers are increasingly concerned about the quality and safety of many products present in the diet of industrialized countries, in particular, the use of artificial sweeteners, flavorings, colorings, preservatives, and dietary supplements. General apprehension also exists regarding the possible long-term health effects of the raw materials and technologies used for the packaging, sterilization, and distribution of foods. Of particular concern are the potential carcinogenic effects of these products and processes.

The experimental and epidemiologic data currently available to evaluate the above carcinogenic risks are insufficient and often unreliable because of the inadequate planning and conduct of previous experiments. This inadequacy, combined with the general limited knowledge about the safety and potential carcinogenic effects of substances widely present in the industrialized diet, motivated the design of an integrated project of mega-experiments in 1985 at the Cesare Maltoni Cancer Research Center (CMCRC) of the European Ramazzini Foundation (ERF). The products studied are reported in Table 1. The products and agents we selected for this project were those for which committee debate and opinions had often acted as surrogates for good laboratory work. At present, over the course of the project, 32 long-term bioassays have been performed using > 25,000 rodents. Studies have evaluated the carcinogenicity of 12 different products, including the artificial sweetener aspartame (APM).

In this article we present the results of the mega-experiment on the carcinogenicity of APM in which the sweetener was administered in feed to Sprague-Dawley rats for the life span.

APM, the methyl ester of the dipeptide l-α-aspartyl-l-phenylalanine (C_14_H_18_N_2_O_5_), is a widely used artificial sweetener with a molecular weight of 294.3. Under particular conditions (extreme pH, high temperature, lengthy storage times), APM may be contaminated by the diketopiperazine (DKP) cyclo-aspartylphenylalanine (Butchko et al. 2002a).

For more than 30 years, APM has been widely used as a food additive because of its very strong, sweet taste. The sweetening power of APM is estimated to be 200 times that of sucrose, whereas saccharin and cyclamate are 300 and 30 times sweeter, respectively (Mazur 1984).

Initial commercial approval of APM in the United States was granted by the Food and Drug Administration (FDA 1974). The FDA later approved the limited use of APM in solid foods in 1981 and extended this authorization to soft drinks in 1983. APM was eventually approved as a general sweetener in 1996 (FDA 1981, 1983, 1996). In the European Union, the safe use of APM was authorized in 1994 (EC Directive 1994).

After saccharin, APM is the second most used artificial sweetener in the world. It is estimated that > 8,000 tons of APM are consumed each year in the United States (Hazardous Substances Data Bank 2005). In terms of world consumption, APM represents 62% of the value of the intense sweetener market (Fry 1999).

APM is found in > 6,000 products, including carbonated and powdered soft drinks, hot chocolate, chewing gum, candy, desserts, yogurt, tabletop sweeteners, and some pharmaceutical products, such as vitamins and sugar-free cough drops, and is estimated by the Aspartame Information Center (2005) to be consumed by > 200 million people worldwide.

Through dietary surveys performed in the United States among APM consumers during the period 1984–1992, the average APM daily intake in the general population has been shown to range from 2 to 3 mg/kg body weight (bw). Consumption by children 2–5 years of age and by females of childbearing age in these surveys ranged from about 2.5 to 5 mg/kg bw/day (Butchko et al. 2002b). APM intake was also monitored in several other regions, including seven European countries. Although survey methodologies may have differed, the APM intake was remarkably consistent across studies and was well below the acceptable daily intake (ADI) both in the United States (50 mg/kg bw) and in Europe (40 mg/kg bw) (Butchko et al. 2002b).

Investigations into the metabolism of APM have shown that, in rodents, nonhuman primates, and humans, it is metabolized in the gastrointestinal tract into three constituents—aspartic acid, phenylalanine, and methanol—which are absorbed into the systemic circulation (Ranney et al. 1976). For each molecule of APM, one molecule of each constituent is produced. After absorption, they are then used, metabolized, and/or excreted by the body following the same metabolic pathways as when consumed through the ordinary diet: aspartate is transformed into alanine plus oxaloacetate (Stegink 1984); phenylalanine is transformed mainly into tyrosine and, to a smaller extent, phenylethylamine and phenyl-pyruvate (Harper 1984); and methanol is transformed into formaldehyde and then to formic acid (Opperman 1984).

APM was not genotoxic in the following tests: dominant lethal mutation assay in rats, host-mediated assay in rats and mice, *in vivo* cytogenetic assay in rats, and the Ames test (Kotsonis and Hjelle 1996). Results of an assay to measure induction of unscheduled DNA synthesis in rat hepatocytes treated with APM *in vitro* were negative, indicating the absence of APM-induced DNA damage (Jeffrey and Williams 2000). In a test for the induction of chromosomal aberration in bone marrow cells of male Swiss mice, Mukhopadhyay et al. (2000) reported that a mixture of APM (up to 350 mg/kg) and a second sweetener, acesul-fame potassium (up to 150 mg/kg) administered by gavage was negative. However, a dose-related increase in the percentage of cells with chromosomal aberrations was noted with increasing doses of the two sweeteners, even though the increase was not statistically significant (Mukhopadhyay et al. 2000).

Two long-term feeding carcinogenicity bioassays on APM were performed on rats and one on mice in the early 1970s by the producer Searle & Co. Results were reviewed by the FDA and then summarized in the *Federal Register* (FDA 1981). To date, the details of the experiments have not been published.

In the first study, groups of 40 male and 40 female Sprague-Dawley rats were treated with 1, 2, 4, or 6–8 g/kg bw/day of APM in the diet. The treatment started at 4 weeks of age and lasted for a period of 104 weeks. A control group of 60 rats per sex was fed the same diet without APM. At the end of the treatment, all surviving animals were sacrificed and their brains, as well as other organs (not specified in the report), were examined histologically. Brain tumors were observed in 7 of 155 (4.5%) exposed males versus 1 of 59 (1.7%) controls, and in 5 of 158 (3.2%) exposed females versus 0 of 59 (0%) controls. Overall, the FDA considered the study to be negative with regard to the carcinogenicity of APM (FDA 1981).

In the second study, groups of 40 male and 40 female Sprague-Dawley rats were exposed to APM, at doses of 2 and 4 g/kg bw/day, through their mothers’ diet both *in utero* and during lactation, and then for 104 weeks with APM in their own diets. A control group of 60 rats per sex was fed the same diet without APM. The animals were necropsied at the time of death or at 104 weeks after weaning. Three brain tumors were observed among control males and one among control females. Brain tumors were also observed in two males and one female in the 2 g/kg bw group, and in one male and one female in the 4 g/kg bw group. Again, the FDA considered the study to be negative with regard to the carcinogenicity of APM (FDA 1981).

Regarding the third chronic APM study, in this case performed on mice, the FDA reported that the results did not show any treatment-related carcinogenic effect. In this experiment, as reported by Molinary (1984), groups of 36 male and 36 female mice were fed 1, 2, or 4 g/kg bw/day until 110 weeks of age. A group of 72 males and 72 females served as the control. There were no treatment-related effects on survival and behavior, nor were any lesions recorded during macroscopic or microscopic analysis.

An APM carcinogenicity study was also conducted in Japan during this period (Ishii 1981; Ishii et al. 1981). Groups of 86 male and 86 female Wistar rats were treated with APM in feed at doses of 0, 1, 2, or 4 g/kg bw/day from 6 to 110 weeks of age. No increase in the incidence of brain tumors was observed in the treated groups compared with the controls. Exhaustive experimental details of this study were not published.

Epidemiologic studies to evaluate the relationship between APM intake and cancer development in humans are not currently available.

Although all of the aforementioned studies were considered negative with respect to the carcinogenicity of APM, in our opinion, these studies did not comply with today’s basic requirements for testing the carcinogenic potential of a physical or chemical agent, in particular concerning the number of animals for each experimental group and the duration of the experiment until 110 weeks of age of the animals.

For these reasons, and in light of the ever-increasing diffusion of APM in the diet of industrialized countries (particularly in products consumed by young children and pregnant women), we considered it important to perform a mega-experiment following today’s internationally recognized good laboratory practices for carcinogenicity bioassays and, more specifically, the life-span carcinogenicity bioassay design followed for many years at the CMCRC and described in previous publications (Soffritti et al. 1999, 2002c).

## Materials and Methods

APM, as a food-grade material, was produced by Nutrasweet and supplied by Giusto Faravelli S.p.A. (Milan, Italy). Its purity was > 98%: DKP was < 1.5% and l-phenylalanine was < 0.5%. An infrared absorption spectrophotometer assay was used to determine APM purity. An assumed daily intake by humans of 5,000, 2,500, 500, 100, 20, 4, or 0 mg/kg bw was simulated by adding APM to the standard Corticella diet (Laboratori Dottori Piccioni, Milan Italy), used for 30 years at the CMCRC/ERF laboratory, at concentrations of 100,000, 50,000, 10,000, 2,000, 400, 80, or 0 ppm. The APM daily assumption in milligrams per kilogram body weight was calculated considering the average weight of a rat for the duration of the experiment as 400 g, and the average consumption of feed as 20 g/day, both for males and females. APM was administered with feed *ad libitum* to Sprague-Dawley rats (100–150/sex/group). The experiment started when the animals were 8 weeks of age, and the treatment lasted until natural death. Control animals received the same feed without APM. The experiment was conducted according to Italian law regulating the use of animals for scientific purposes (Decreto Legislativo 116 1992), which provides the guidelines on how to treat animals humanely and without suffering.

Rodents used for the experiment were male and female Sprague-Dawley rats from the colony of the CMCRC/ERF. This colony of rats has been employed for various experiments in the laboratory for nearly 30 years, and extensive historical data are available on the tumor incidence among untreated rats. All control animals were monitored for feed and water consumption and body weight for their life span and, upon death, underwent complete necropsy and histopathologic evaluation.

The health status of the animals was regularly checked by the veterinarians of the local and national health services. Before matching, the breeders were clinically observed for their health status, in order to exclude any diseased animals, and the experimental animals were clinically examined monthly until the end of the experiment.

At 4–5 weeks of age, after weaning, the experimental animals were randomized in order to have no more than one male and one female from each litter in the same group. They were then housed, in groups of five, in Makrolon cages (41 cm × 25 cm × 15 cm), with stainless-steel wire tops and a shallow layer of white wood shavings as bedding, and kept in rooms used only for this experiment, at a temperature of 23 ± 2°C and relative humidity of 50–60%.

Once a week for the first 13 weeks, then every 2 weeks until the rats were 110 weeks of age, the mean daily drinking water and feed consumption was measured per cage, and body weight was measured individually. Measurement of body weight continued every 8 weeks until the end of the experiment. The animals were clinically examined for gross changes every 2 weeks for the duration of the experiment. To evaluate the status and behavior of the animals and to limit the postmortem modifications (pmm), a patrol was performed three times daily from Monday through Friday and twice on Saturdays and Sundays and holidays. Dead animals were registered and kept refrigerated at 4°C until necropsy. Based on this procedure [part of our longstanding standard operating procedures (SOP)], very few animals were affected by pmm, and only on very rare occasions did this interfere with the ability to histologically diagnose and interpret some lesions.

The biophase ended at 151 weeks, with the death of the last animal at the age of 159 weeks. Upon death, the animals underwent complete necropsy. Histopathology was routinely performed on the following organs and tissues of each animal from each group: skin and subcutaneous tissue, mammary gland, the brain (three sagittal sections), pituitary gland, Zymbal glands, salivary glands, Harderian glands, cranium (five sections, with oral and nasal cavities and external and internal ear ducts), tongue, thyroid, parathyroid, pharynx, larynx, thymus and mediastinal lymph nodes, trachea, lung and mainstem bronchi, heart, diaphragm, liver, spleen, pancreas, kidneys, adrenal glands, esophagus, stomach (fore and glandular), intestine (four levels), urinary bladder, prostate, gonads, interscapular brown fat pad, subcutaneous and mesenteric lymph nodes, and other organs or tissues with pathologic lesions. All organs and tissues were preserved in 70% ethyl alcohol, except for bones, which were fixed in 10% formalin and then decalcified with 10% formaldehyde and 20% formic acid in water solution. The normal specimens were trimmed following the CMCRC/ERF laboratory SOP. Trimmed specimens were processed as paraffin blocks, and 3–5 μm sections of every specimen were obtained.

Sections were routinely stained with hematoxylin and eosin (H&E). Immunohistochemical staining for S100 was performed to characterize malignant schwannoma, whereas chromogranin A staining was used to characterize olfactory neuroblastomas. For S100 staining, we used a polyclonal rabbit anti-S100 (Z0311; Dakocytomation, Carpinteria, CA, USA) as primary antibody, whereas for chromogranin A staining, we used a polyclonal rabbit anti-human chromogranin A (N1535, Dakocytomation) (Information Center for Immunohistochemistry 2005).

Two statistical tests were used to analyze neoplastic and nonneoplastic lesion incidence data. We used the Cochran-Armitage trend test (Armitage 1971; Gart et al. 1979) to test for linear trends in tumor incidence. We also used the poly-*k* test (Bailer and Portier 1988; Piegorsch and Bailer 1997; Portier and Bailer 1989), a survival-adjusted quantal response modification of the Cochran-Armitage test that takes survival into account. The tests used and the resulting *p*-values are reported in the tables.

## Results

**Figure 1:**
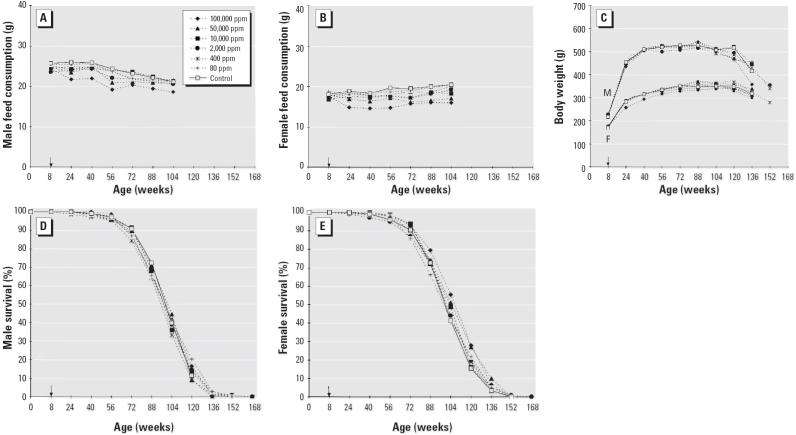
. Comparison of untreated and treated male and female rats. (*A*) Mean daily feed consumption in males. (*B*) Mean daily feed consumption in females. (*C*) Mean body weights in males (M) and females (F). (*D*) Survival in males. (*E*) Survival in females. The arrow indicates the start of the experiment at 8 weeks of age.

The study proceeded smoothly without unexpected occurrences. We observed no differences in water consumption between the treated and the untreated groups, whereas a dose-related difference in feed consumption was observed between the various treated groups and the control group in both males and females (Figure 1A,B). No substantial differences in mean body weight were observed between the treated and control groups, apart from a slight decrease in females treated at 100,000 ppm APM (Figure 1C). No substantial difference in survival was observed among the groups (Figure 1D,E).

No evident behavioral changes were observed among treated animals compared with controls. In animals exposed to the highest dose of APM, yellowing of the coat was observed; this change had previously been observed in our laboratory in rats exposed to formaldehyde administered with drinking water (Soffritti et al. 2002b).

The carcinogenic effects of APM are reported in Table 2 for males and Table 3 for females. Multiple tumors of different types and sites; of different types in the same site; of the same types in bilateral organ; of the same types in the skin, subcutaneous tissue, or mammary glands; or at distant sites of diffuse tissue (i.e., bones and skeletal muscle) were plotted as single/independent tumors. Multiple tumors of the same type in the same tissue and organ, apart those above mentioned, were plotted only once.

### Total malignant tumors.

The incidence of animals bearing malignant tumors occurred with a significant positive trend in males (*p* ≤ 0.05) and in females (*p* ≤ 0.01), as reported in Tables 2 and 3. A statistically significant increase of the incidence of malignant tumors was observed in females treated at 50,000 ppm (*p* ≤ 0.01) compared with the control group (Table 3). Tumor types that contributed most are presented below.

### Lymphomas/leukemias.

The data on the occurrence of lymphomas/leukemias, reported in Tables 2 and 3, indicate that APM causes a significant positive trend in males (*p* ≤ 0.05) and in females (*p* ≤ 0.01). Compared with untreated control groups, the increased incidence of lymphomas/leukemias in treated females was statistically significant at doses of 100,000 (*p* ≤ 0.01), 50,000 (*p* ≤ 0.01), 10,000 (*p* ≤ 0.05), 2,000 (*p* ≤ 0.05), or 400 ppm (*p* ≤ 0.01). The most frequent histocytotypes observed in the experiment were lymphoimmunoblastic lymphomas, mainly involving lung and mediastinal/peripheral nodes, and histiocytic sarcomas, involving mainly lung, liver, spleen, and nodes. The distribution of lymphomas/leukemias by histocytotypes is presented in Table 4. The differential diagnoses were based on the morphologic criteria followed in our laboratory for several decades and are in line with the guidelines of the International Classification of Rodent Tumors [International Agency for Research on Cancer (IARC) 1993]. Lymphomas/leukemias (this term includes all types of hemolymphosarcomas and leukemias) are neoplasias arising from hemolymphoreticular tissues, and their aggregation is widely used in experimental carcinogenesis because both solid and circulating phases are present in many lymphoid neoplasms, and distinction between them is artificial (Harris et al. 2001).

### Preneoplastic and neoplastic lesions of the renal pelvis and ureter.

**Figure 2:**
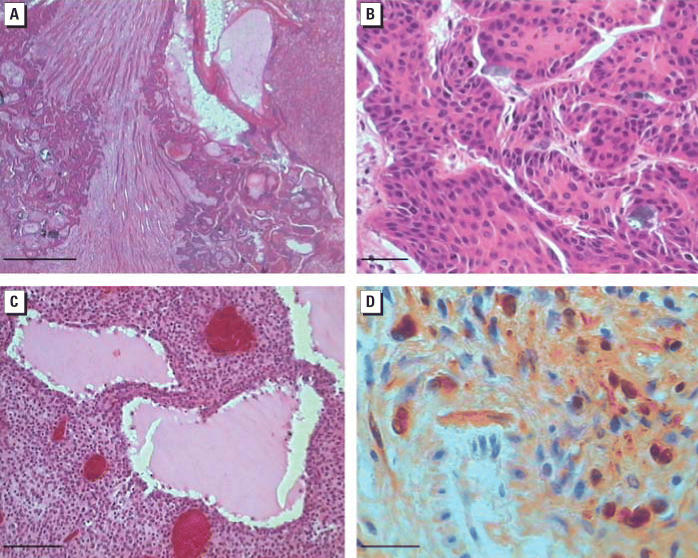
(*A*) Carcinoma of the renal pelvis in a female rat administered 100,000 ppm APM in feed; H&E; magnification, 25×; bar = 500 μm. (*B*) Detail of the carcinoma shown in (*A*); H&E; magnification, 400×; bar = 20 μm. (*C*) Malignant schwannoma of cranial nerves resembling Antoni B type pattern in a male rat administered 100,000 ppm APM in feed; H&E; magnification, 200×; bar = 50 μm. (*D*) Immunohistochemical characterization with S100 protein of the schwannoma shown in (*C*); magnification, 1,000×; bar = 10 μm.

The incidences of pre-neoplastic and neoplastic lesions of the transitional cell epithelium of the renal pelvis and ureter are reported in Tables 2 and 3. A dose-related increase in the incidence of dysplastic hyperplasias and dysplastic papillomas of the renal pelvis and ureter was observed in females. Carcinomas in females occurred with a positive trend (*p* ≤ 0.05), and the incidence in females exposed at 100,000 ppm was significantly higher (*p* ≤ 0.05) compared with the controls. Carcinomas were also observed among males treated at 100,000, 50,000, 10,000, or 2,000 ppm. In females, dysplastic lesions and carcinomas combined show a significant positive trend (*p* ≤ 0.01) and a statistically significant increase in those treated at 100,000 (*p* ≤ 0.01), 50,000 (*p* ≤ 0.01), 10,000 (*p* ≤ 0.01), 2,000 (*p* ≤ 0.05), or 400 ppm (*p* ≤ 0.05). A 3-fold increase is also observed in the group treated with 80 ppm. We did not observe substantial differences in the incidence of inflammation between males and females treated at the different doses, compared with controls. Increased incidence of calcification was observed in females, particularly in those treated at 100,000 ppm (39%), 50,000 ppm (25%), or 10,000 ppm (19%), compared with controls (8%); this effect was not observed in males. Although transitional cell carcinomas of the renal pelvis and ureter are extremely rare in male and female untreated rats, the APM male and female groups had a total of 21 transitional cell carcinomas of the renal pelvis, whereas the controls had none. Microscopically, the carcinomas were invading, with various levels of extension, the papilla and the kidney parenchyma; the cells were of transitional type, and several mitotic figures were present (Figure 2A,B).

### Malignant schwannomas of peripheral nerves.

As shown in Table 2, the incidence of malignant schwannomas of the peripheral nerves occurred with a positive trend (*p* ≤ 0.05) in males. In females, nine malignancies were observed among treated animals of the different dosage groups, and none among the controls (Table 3). All lesions, in males and females, diagnosed as malignant schwannomas were positive for S100 staining. The most frequent site of origin of the malignant schwannomas was in the cranial nerves (72%); the other cases arose at the spinal nerve roots. Microscopically, malignant schwannomas invaded the soft tissues locally. Metastases of cranial nerve malignant schwannomas were observed in three males treated at the highest dose. The metastases were found in submandibular lymph nodes in two cases, and the tumor metastatized to the lung and liver in one case. Histologically the feature of malignant schwannomas was Antoni B type (Figure 2C,D).

### Preneoplastic and neoplastic lesions of the olfactory epithelium.

Incidence of hyperplasia of the olfactory epithelium increased with a significant positive trend in males and females. The observed incidences were, respectively, 14.0% and 18.0% in males and females exposed at 100,000 ppm, 12.0% and 21.0% at 50,000 ppm, 7.0% and 17.0% at 10,000 ppm, 2.7% and 8.7% at 2,000 ppm, 6.0% and 7.3% at 400 ppm, 2.0% and 3.3% at 80 ppm, and 0.7% and 4.0% at 0 ppm. The differences were statistically significant (*p* ≤ 0.01) at 100,000, 50,000, or 10,000 ppm in both males and females and at 400 ppm in males. Among females treated at the highest dose, one case of dysplastic hyperplasia, one adenoma, and one olfactory neuroblastoma were observed. The neuroblastoma invaded the cranium, compressing the forebrain, and was positive for chromogranin A immunohistochemical staining.

### Malignant brain tumors.

Concerning the incidence of malignant tumors in the brain, it should be noted that, as previously reported (Soffritti et al. 2005), 12 malignant tumors (10 gliomas, 1 medulloblastoma and 1 meningioma) were observed, without dose relationship, in male and female APM-treated groups, whereas none were observed in controls.

### Other malignant tumors.

The other malignant tumors were among those commonly observed in Sprague-Dawley rats, apart from two transitional cell carcinomas of the bladder observed in males exposed to 10,000 ppm, one in females exposed to 2,000 ppm, and none among the controls. Because this type of tumor is extremely rare among the historical controls of our colony of Sprague-Dawley rats, this occurrence cannot be disregarded.

### Historical controls.

Over the last 20 years in our laboratory, when we consider only groups of > 100 animals/sex, the numbers of the untreated males and females total 1,934 and 1,945 respectively. Concerning the renal pelvis and ureter transitional cell carcinomas, no carcinomas were observed in either males or females. The overall incidence of malignant schwannomas was 0.5% (range, 0–2.0%) in males and 0.1% (range, 0–1.0%) in females. The overall incidence of lymphomas/leukemias was 20.7% (range, 8.0–30.9%) in males and 12.4% (range, 7.0–18.4%) in females. The overall incidence of olfactory neuroblastoma was 0.1% (0–1.8%) in both males and females.

When we also consider control groups of < 100 animals/sex, the numbers of untreated males and females total 2,265 and 2,274, respectively. The overall incidence of the renal pelvis and ureter transitional cell carcinomas was 0.04% (range, 0–1.0%) in females, whereas no carcinomas were observed in males. The overall incidence of malignant schwannomas was 0.4% (range, 0–2.0%) in males and 0.1% (range, 0–2.0%) in females. The overall incidence of lymphomas/leukemias was 20.6% (range, 8.0–30.9%) in males and 13.3% (range, 4.0–25.0%) in females. The overall incidence of olfactory neuroblastomas was 0.1% (range, 0–1.8%) in both males and females.

## Discussion

The mega-experiment performed in our laboratory on APM (administered with feed to Sprague-Dawley rats from 8 weeks of age until natural death) has shown for the first time the multipotential carcinogenic effects of this compound. In fact, the results indicate that APM causes, in our experimental conditions, *a*) an increased incidence of malignant-tumor–bearing animals with a positive significant trend in males (*p* ≤ 0.05) and in females (*p* ≤ 0.01), particularly in the females treated at 50,000 ppm (*p* ≤ 0.01); *b*) a statistically significant dose-related increase of the incidence of lymphomas/leukemias in females treated at the doses of 100,000 (*p* ≤ 0.01), 50,000 (*p* ≤ 0.01), 10,000 (*p* ≤ 0.05), 2,000 (*p* ≤ 0.05), or 400 ppm (*p* ≤ 0.01) and a positive significant trend in both males (*p* ≤ 0.05) and females (*p* ≤ 0.01); *c*) in females, dysplastic lesions and carcinomas of the renal pelvis and ureter combined show a significant positive trend (*p* ≤ 0.01) and a statistically significant increase in those treated at 100,000 (*p* ≤ 0.01), 50,000 (*p* ≤ 0.01), 10,000 (*p* ≤ 0.01), 2,000 (*p* ≤ 0.05), or 400 ppm (*p* ≤ 0.05); and *d*) an increased incidence of malignant schwannomas of the peripheral nerves with a positive trend (*p* ≤ 0.05) in males.

The increase in lymphomas/leukemias in APM-treated females could be related to its metabolite methanol, which is in turn metabolized to formaldehyde in both humans and rats (Ranney et al. 1976). In fact, previous experiments performed at the CMCRC laboratory have shown that *a*) methanol administered in drinking water, at doses ranging from 20,000 to 500 ppm, induced a statistically significant increase in the incidence of lymphomas/leukemias in female rats (Soffritti et al. 2002a); *b*) a dose-related increase in the incidence of lymphomas/leukemias was also observed in females treated with formaldehyde, administered in drinking water at doses ranging from 1,500 to 50 ppm (Soffritti et al. 1989, 2002b); and *c*) the same effect was observed in females treated with the gasoline oxygenated additive methyl-*tert*-butyl ether (MTBE), which metabolizes to methanol (Belpoggi et al. 1995).

The important role of formaldehyde in the induction of hematologic malignancies in rodents is further highlighted by these results. In a recent reevaluation of the carcinogenicity of formaldehyde by the IARC (in press), strong (although not considered sufficient) evidence of an association between formaldehyde exposure and leukemias in humans was found.

Moreover, carcinogenic effects for the renal pelvis and ureter, peripheral nerves and proliferative changes of the olfactory epithelium were not observed in the long-term bioassays performed in the same conditions at the CMCRC on methanol, MTBE, or formaldehyde. To investigate if the other two metabolites of APM are responsible for inducing these lesions, it is of paramount importance to perform adequate life-span carcinogenicity studies on aspartic acid or phenylalanine.

In a long-term carcinogenicity study on monosodium aspartate (MSA) administered with drinking water to groups of 50 male and 50 female Fischer-344 rats (beginning at 6 weeks of age for 100 weeks and then sacrificed), a dose-related hyperplasia of the renal pelvis was observed in males and in females (Kitahori et al. 1996). The same effect was found by the same group of investigators in another study in which MSA was administered in drinking water to groups of male and female Fischer-344 rats to evaluate its promoting activity of carcinogenesis of the transitional epithelium of the renal pelvis (Kitamura et al. 1996). In both studies, clear evidence was provided of a relationship between MSA treatment and transitional cell hyperplasia. The authors indicated that calcification could have an important role in inducing simple and papillary hyperplasia of the renal pelvis transitional cell epithelium and, consequently, in the induction of transitional cell tumors. In our study performed on 1,800 Sprague-Dawley rats, which are less susceptible to the spontaneous development of nephropathies than Fischer rats, we observed a dose-related, statistically significant increase in the incidence of dysplastic hyperplasia and carcinoma of the renal pelvis in females, but none in males, compared with the controls. The fact that we observed an increased incidence of kidney calcification in females and not in males, compared with the controls, gives added weight to the hypothesis that aspartic acid may cause pre-neoplastic and neoplastic lesions of the renal pelvis, and that calcification may be the mechanism responsible for this effect.

The carcinogenic effects of APM observed in our experiment are in contrast with the results obtained with long-term carcinogenicity bioassays, performed almost 30 years ago on Sprague-Dawley rats, which did not reveal APM to have any carcinogenic effects (FDA 1981). There are several reasons that can explain this difference. First of all, in our experiment the number of animals per sex per group was much greater, allowing a more thorough and reliable statistical analysis. Second, in our experiment, rodents were not killed at 110 weeks of age but rather were observed until natural death, to allow APM to fully express its carcinogenic potential. Had we stopped the experiments at 110 weeks of age, we would most likely never have demonstrated the carcinogenicity of important industrial compounds such as xylenes, mancozeb, vinyl acetate monomer (Soffritti et al. 2002c), and toluene (Soffritti et al. 2004).

Finally, concerning the absence of carcinogenic effects observed in the experiment performed on Wistar rats (Ishii 1981; Ishii et al. 1981), it cannot be disregarded that this strain is more resistant than Sprague-Dawley rats to developing cancer, a characteristic shown in our experiments on benzene (Maltoni et al. 1989). Moreover, the aforementioned experiment on Wistar rats was terminated at the age of 110 weeks. Given these differences, the results of the Wistar rat study are not comparable with those performed on Sprague-Dawley rats.

## Conclusions

Our study shows that APM is a multi-potential carcinogenic compound whose carcinogenic effects are evident even at a daily dose of 20 mg/kg bw, much less than the current ADI for humans in Europe (40 mg/kg bw) and in the United States (50 mg/kg bw).

The results of carcinogenicity bioassays in rodents are consistent predictors of human cancer risks (Huff 1999; Rall 1995; Tomatis et al. 1989). The results of our study therefore call for an urgent reexamination of the present guidelines on the use and consumption of APM. The decision to use experimental data to protect public health is important because the time span of widespread APM use is still too brief to have produced solid epidemiologic data. Moreover, it is unlikely that sufficient epidemiologic data will be available in the near future, given the difficulty of finding a control group that has not been exposed to this widely diffused compound.

## Figures and Tables

**Table 1:** Beverages and diet products studied at the CMCRC/ERF: status of studies.

			Animals		
No.	Products	No. of bioassays	Species	No.	Study status
1	Water in polyvinyl chloride bottles	2	Rat^a^	2,200	P^b^
2	Coca-Cola	4	Rat^a^	1,999	RP
3	Pepsi Cola	1	Rat	400	E
4	Ethyl alcohol (10% vol/vol)	4	Rat,^a^ mouse	1,458	P^c^
5	Sucrose	1	Rat	400	E
6	APM	6	Rat, mouse^a^	4,460	BO, PP^d^
7	Sucralose	1	Mouse^a^	760	BO
8	Caffeine	1	Rat	800	E
9	Vitamin A	5	Rat	5,100	PP^e^
10	Vitamin C	5	Rat	3,680	E
11	Vitamin E	5	Rat	3,680	E
12	Feed sterilized by gamma radiation	1	Rat^a^	2,000	E
Total		36		26,937	

Abbreviations: BO, biophase ongoing; E, in elaboration; P, published; PP, partially published; RP, ready for publication.

a Treatment started from embryonic life.

b Data from Maltoni et al. (1997).

c Data from Soffritti et al. (2002a).

d Data from Soffritti et al. (2005).

e Data from Soffritti et al. (1992).

**Table 2:** Incidence of the preneoplastic and neoplastic lesions in male Sprague-Dawley rats in a life-span feed carcinogenicity study of APM.

		Malignant tumors^a^						Animals bearing dysplastic lesions and carcinomas of the renal pelvis and ureter^a^								Animals bearing peripheral nerve malignant schwannomas^a^					
		Tumor-bearing animals^b^		Total tumors		Total animals bearing lymphomas/leukemias^a^,^b^		Dysplastic hyperplasias		Dysplastic papillomas		Carcinomas		Total		Cranial		Other sites		Total^b^	
Dose, ppm (mg/kg bw)	Animals at start	No.	%	No.	Per 100 animals	No.	%	No.	%	No.	%	No.	%	No.	%	No.	%	No.	%	No.	%
100,000 (5,000)	100	43	43.0	55	55.0	29	29.0	3	3.0	0	—	1	1.0	4	4.0	3	3.0	1	1.0	4	4.0
50,000 (2,500)	100	38	38.0	45	45.0	20	20.0	2	2.0	0	—	1	1.0	3	3.0	3	3.0	0	—	3	3.0
10,000 (500)	100	34	34.0	42	42.0	15	15.0	2	2.0	0	—	1	1.0	3	3.0	2	2.0	0	—	2	2.0
2,000 (100)	150	60	40.0	69	46.0	33	22.0	4	2.7	0	—	1	0.7	5	3.3	2	1.3	0	—	2	1.3
400 (20)	150	48	32.0	52	34.7	25	16.7	4^c^	2.7	1^c^	0.7	0^c^	—	5^c^	3.4	1	0.7	2	1.3	3	2.0
80 (4)	150	44	29.3	49	32.7	23	15.3	3^c^	2.0	0^c^	—	0^c^	—	3^c^	2.0	1	0.7	0	—	1	0.7
0 (0)	150	53	35.3*	59	39.3	31	20.7*^,#^	1	0.7	0	—	0	—	1	0.7	1	0.7	0	—	1	0.7*^,#^

a The tumor rates are based on the number of animals examined (necropsied).

b *p*-Values associated with the trend test are near the control incidence.

c Tissues from 149 animals were analyzed.

* Statistically significant (*p* ≤ 0.05) using Cochran-Armitage test.

# Statistically significant (*p* ≤ 0.05) using poly-*k* test (*k* = 3).

**Table 3:** Incidence of the preneoplastic and neoplastic lesions in female Sprague-Dawley rats in a life-span feed carcinogenicity study of APM.

		Malignant tumors^a^						Animals bearing dysplastic lesions and carcinomas of the renal pelvis and ureter^a^,^b^,^c^								Animals bearing peripheral nerve malignant schwannomas^a^					
		Tumor-bearing animals^b^,^c^		Total tumors		Total animals bearing lymphomas/leukemias^a^,^b^,^c^		Dysplastic hyperplasias		Dysplastic papillomas		Carcinomas^d^		Total		Cranial		Other sites		Total^b^	
Dose, ppm (mg/kg bw)	Animals at start	No.	%	No.	Per 100 animals	No.	%	No.	%	No.	%	No.	%	No.	%	No.	%	No.	%	No.	%
100,000 (5,000)	100	51	51.0	64	64.0	25	25.0##	8	8.0	3	3.0	4	4.0#	15	15.0##	1	1.0	1	1.0	2	2.0
50,000 (2,500)	100	58	58.0##	84	84.0	25	25.0##	6^e^	6.1	1^e^	1.0	3^e^	3.0	10^e^	10.1##	1	1.0	0	—	1	1.0
10,000 (500)	100	40	40.0	62	62.0	19	19.0#	6	6.0	1	1.0	3(4)	3.0	10	10.0##	1	1.0	0	—	1	1.0
2,000 (100)	150	67	44.7	86	57.3	28	18.7#	6	4.0	1	0.7	3(4)	2.0	10	6.7#	1	0.7	2	1.3	3	2.0
400 (20)	150	70	46.7	95	63.3	30	20.0##	5	3.3	1	0.7	3	2.0	9	6.0#	0	—	0	—	0	—
80 (4)	150	64	42.7	85	56.7	22	14.7	4	2.7	1	0.7	1	0.7	6	4.0	1	0.7	1	0.7	2	1.3
0 (0)	150	55	36.7**	69	46.0	13	8.7**^,#^	2	1.3**	0	—*	0	—	2	1.3**^,##^	0	—	0	—	0	—

a The tumor rates are based on the number of animals examined (necropsied).

b *p-*Values corresponding to pairwise comparisons between the controls and the dosed group are near the dosed group incidence .

c *p*-Values associated with the trend test are near the control incidence.

d Values in parentheses indicate the number of tumors (one animal can bear bilateral tumors).

e Tissues from 99 animals were analyzed.

* Statistically significant (*p* ≤ 0.05) using Cochran-Armitage test.

** Statistically significant (*p* ≤ 0.01) using Cochran-Armitage test.

# Statistically significant (*p* ≤ 0.05) using poly-*k* test (*k* = 3).

## Statistically significant (*p* ≤ 0.01) using poly-*k* test (*k* = 3).

**Table 4:** Incidence and distribution by hystocytotype of lymphomas/leukemias in female Sprague-Dawley rats in a life-span feed carcinogenicity study of APM.

				Lymphomas/leukemias^a^															
		Total lymphomas/leukemias^b^		Lymphoblastic lymphoma		Lymphoblastic leukemia		Lymphocytic lymphoma		Lymphoimmunoblastic lymphoma		Histiocytic sarcoma		Monocytic leukemia		Myeloid leukemia	
Dose, ppm (mg/kg bw)	Animals at start	No.	%	No.	%	No.	%	No.	%	No.	%	No.	%	No.	%	No.	%
100,000 (5,000)	100	25	25.0##	1	4.0	0	—	2	8.0	11	44.0	7	28.0	2	8.0	2	8.0
50,000 (2,500)	100	25	25.0##	2	8.0	0	—	0	—	10	40.0	8	32.0	4	16.0	1	4.0
10,000 (500)	100	19	19.0#	2	10.5	0	—	2	10.5	3	15.8	10	52.6	2	10.5	0	—
2,000 (100)	150	28	18.7#	5	17.8	1	3.6	1	3.6	8	28.6	8	28.6	4	14.3	1	3.6
400 (20)	150	30^c^	20.0##	7	23.3	0	—	2	6.7	8	26.7	9	30.0	5	16.7	0	—
80 (4)	150	22	14.7	3	13.6	0	—	5	22.7	6	27.2	6	27.3	2	9.1	0	—
0 (0)	150	13	8.7**^,#^	2	15.4	0	—	2	15.4	5	38.5	4	30.8	0	—	0	—

a Percentage of animals bearing specific histocytotype refer to the total number of animals bearing lymphomas/leukemias.

b Percentage of animals at start to bear lymphomas/leukemias.

c One animal had two types of neoplasias: lymphoblastic lymphoma and histiocytic sarcoma.

** Statistically significant (*p* ≤ 0.01) using Cochran-Armitage test.

# Statistically significant (*p* ≤ 0.05) using poly-*k* test (*k* = 3).

## Statistically significant (*p* ≤ 0.01) using poly-*k* test (*k* = 3).
